# Supplementary figures and images for: Quantifying the effect of Wakefield et al. (1998) on skepticism about MMR vaccine safety in the U.S
Source: PLoS One. 2021 Aug 19;16(8):e0256395. doi: 10.1371/journal.pone.0256395 (PMC8376023; doi:10.1371/journal.pone.0256395)

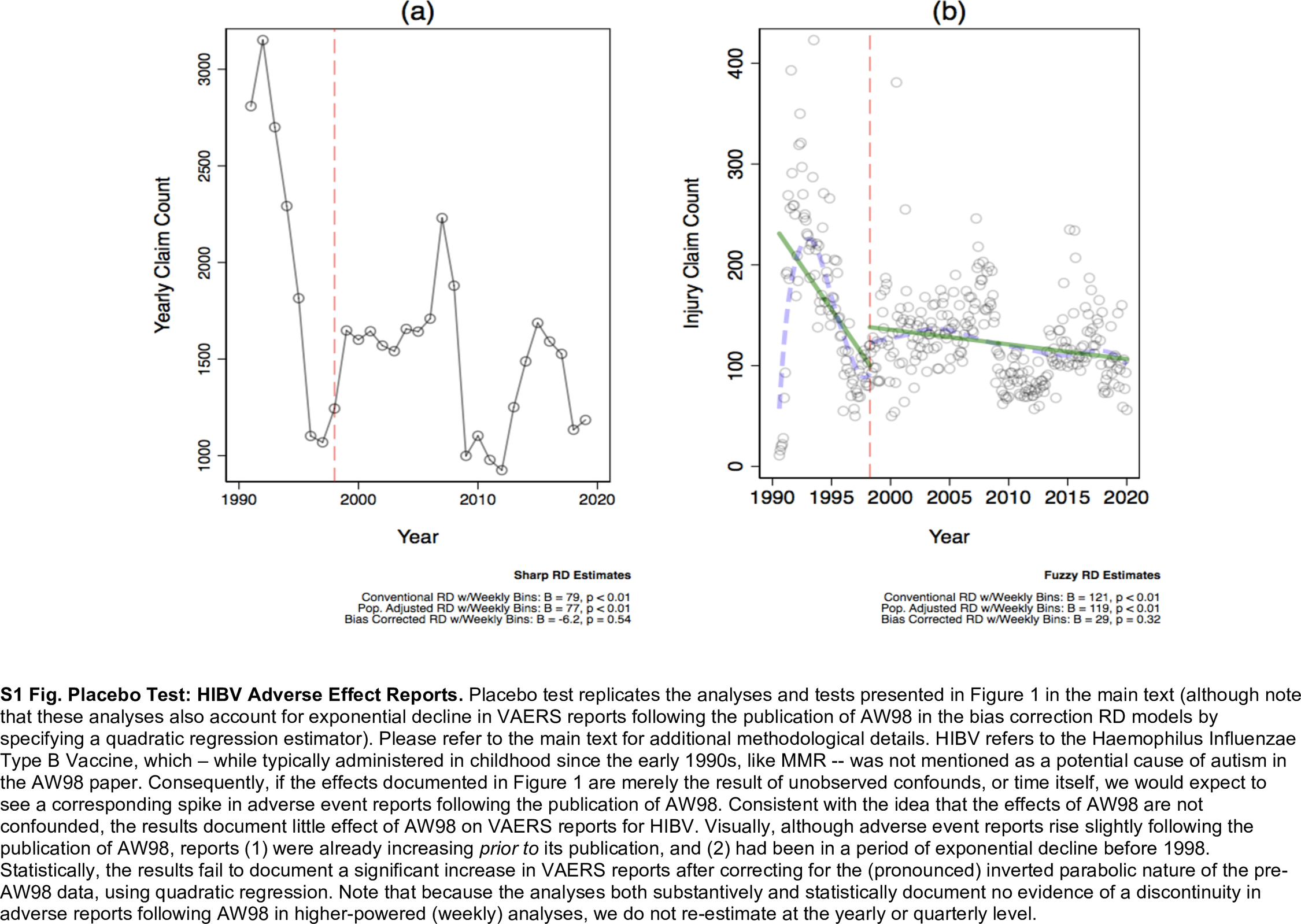

Supplement: S1 Fig — Placebo test replicates the analyses and tests presented in Fig 1 in the main text (although note that these analyses also account for exponential decline in VAERS reports following the publication of AW98 in the bias correction RD models by specifying a quadratic regression estimator). Please refer to the main text for additional methodological details. HIBV refers to the Haemophilus Influenzae Type B Vaccine, which–while typically administered in childhood since the early 1990s, like MMR—was not mentioned as a potential cause of autism in the AW98 paper. Consequently, if the effects documented in Fig 1 are merely the result of unobserved confounds, or time itself, we would expect to see a corresponding spike in adverse event reports following the publication of AW98. Consistent with the idea that the effects of AW98 are not confounded, the results document little effect of AW98 on VAERS reports for HIBV. Visually, although adverse event reports rise slightly following the publication of AW98, reports (1) were already increasing prior to its publication, and (2) had been in a period of exponential decline before 1998. Statistically, the results fail to document a significant increase in VAERS reports after correcting for the (pronounced) inverted parabolic nature of the pre-AW98 data, using quadratic regression. Note that because the analyses both substantively and statistically document no evidence of a discontinuity in adverse reports following AW98 in higher-powered (weekly) analyses, we do not re-estimate at the yearly or quarterly level. (TIF) [file pone.0256395.s001.tif]
